# RETRACTION: Simplified and Modified Limberg Flap Plus Vacuum‐Assisted Closure for Treatment of Sacrococcygeal Pilonidal Sinus Disease

**DOI:** 10.1111/iwj.70596

**Published:** 2025-04-21

## Abstract

**RETRACTION:** XuM. , WangY. , Xi. Ma, LiuY. , XueC. , and DaiH. , “Simplified and Modified Limberg Flap Plus Vacuum‐Assisted Closure for Treatment of Sacrococcygeal Pilonidal Sinus Disease,” International Wound Journal 21, no. 1 (2024): e14353, 10.1111/iwj.14353.37691134 PMC10784422

The above article, published online on 10 September 2023, in Wiley Online Library (http://onlinelibrary.wiley.com/), has been retracted by agreement between the journal Editor in Chief, Professor Keith Harding; and John Wiley & Sons Ltd. Following an investigation by the publisher, all parties have concluded that this article was accepted solely on the basis of a compromised peer review process. The editors have therefore decided to retract the article. The authors did not respond to our notice regarding the retraction.

**RETRACTION:** M. Xu , Y. Wang , Xi. Ma, Y. Liu , C. Xue , and H. Dai , “Simplified and Modified Limberg Flap Plus Vacuum‐Assisted Closure for Treatment of Sacrococcygeal Pilonidal Sinus Disease,” International Wound Journal 21, no. 1 (2024): e14353, 10.1111/iwj.14353.37691134 PMC10784422

The above article, published online on 10 September 2023, in Wiley Online Library (http://onlinelibrary.wiley.com/), has been retracted by agreement between the journal Editor in Chief, Professor Keith Harding; and John Wiley & Sons Ltd. Following an investigation by the publisher, all parties have concluded that this article was accepted solely on the basis of a compromised peer review process. The editors have therefore decided to retract the article. The authors did not respond to our notice regarding the retraction.

